# 2-(Biphenyl-4-yl­oxy)acetic acid

**DOI:** 10.1107/S1600536811002777

**Published:** 2011-01-29

**Authors:** En-Ju Wang, Guang-Ying Chen

**Affiliations:** a Hainan Provincial Key Laboratory of Tropical Pharmaceutical Herb Chemistry, School of Chemistry and Chemical Engineering, Hainan Normal University, Haikou 571158, People’s Republic of China

## Abstract

In the title compound, C_14_H_12_O_3_, the phenyl and benzene rings make a dihedral angle of 47.51 (4)°. In the crystal, mol­ecules are dimerized by double O—H⋯O hydrogen bonds, forming centrosymmetric *R*_2_^2^(8) ring motifs. The dimers are inter­linked by C—H⋯π inter­actions into zigzag layers.

## Related literature

For biological studies of biphenyl compounds, see: Kamoda *et al.* (2006 ▶); Kumar *et al.* (2008 ▶); Malamas *et al.* (2000 ▶). For related structures, see: Ali *et al.* (2008 ▶); Cao (2009 ▶); Margraf *et al.* (2009 ▶); Li *et al.* (2009 ▶); Charbonneau & Delugeard (1977 ▶); Brett *et al.* (1999 ▶). For hydrogen-bond motifs, see: Etter (1990 ▶).
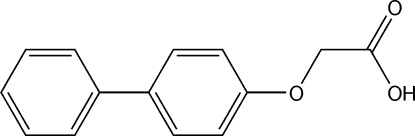


## Experimental

### 

#### Crystal data

C_14_H_12_O_3_
*M* *_r_* = 228.24
Monoclinic, 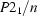

*a* = 5.9118 (1) Å
*b* = 28.5786 (3) Å
*c* = 6.9017 (1) Å
β = 109.631 (2)°
*V* = 1098.27 (3) Å^3^
*Z* = 4
Cu *K*α radiation
μ = 0.79 mm^−1^
*T* = 293 K
0.43 × 0.42 × 0.40 mm

#### Data collection

Bruker SMART CCD area-detector diffractometer
Absorption correction: multi-scan (*SADABS*; Bruker, 2000 ▶) *T*_min_ = 0.727, *T*_max_ = 0.742
11989 measured reflections
2306 independent reflections
2223 reflections with *I* > 2σ(*I*)
*R*_int_ = 0.028

#### Refinement

*R*[*F* ^2^ > 2σ(*F* ^2^)] = 0.047
*wR*(*F* ^2^) = 0.139
*S* = 1.12
2306 reflections
155 parameters
H-atom parameters constrained
Δρ_max_ = 0.21 e Å^−3^
Δρ_min_ = −0.23 e Å^−3^

### 

Data collection: *SMART* (Bruker, 2000 ▶); cell refinement: *SAINT* (Bruker, 2000 ▶); data reduction: *SAINT*; program(s) used to solve structure: *SHELXS97* (Sheldrick, 2008 ▶); program(s) used to refine structure: *SHELXL97* (Sheldrick, 2008 ▶); molecular graphics: *SHELXTL* (Sheldrick, 2008 ▶); software used to prepare material for publication: *SHELXTL*.

## Supplementary Material

Crystal structure: contains datablocks I, global. DOI: 10.1107/S1600536811002777/bh2330sup1.cif

Structure factors: contains datablocks I. DOI: 10.1107/S1600536811002777/bh2330Isup2.hkl

Additional supplementary materials:  crystallographic information; 3D view; checkCIF report

## Figures and Tables

**Table 1:** Hydrogen-bond geometry (Å, °) *Cg* is the centroid of the C9–C14 ring.

*D*—H⋯*A*	*D*—H	H⋯*A*	*D*⋯*A*	*D*—H⋯*A*
O1—H1⋯O2^i^	0.82	1.81	2.6235 (13)	169
C12—H12⋯*Cg*^ii^	0.93	2.86	3.6392 (16)	142

Symmetry codes: (i) 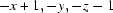; (ii) 
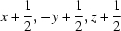
.

**Fig. 1.**
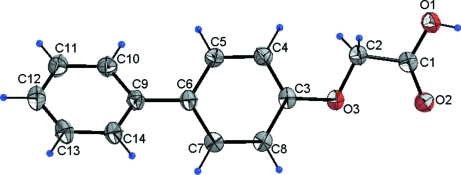
Molecular configuration and atom numbering scheme for the title compound, with displacement ellipsoids drawn at the 50% probability level.

**Fig. 2.**
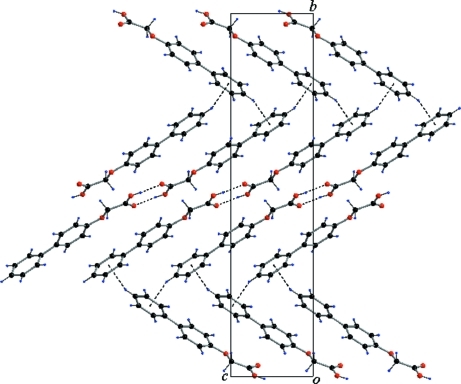
The crystal packing of (I). Hydrogen bonds are shown as dashed lines.

## supplementary crystallographic information

### Comment

Biphenyl moieties have been found to act as pharmacophores in many biological studies such as antimycobacterial testing (Kamoda *et al.*, 2006). Several derivatives of biphenyl-4-yloxy acetic acid are reported to be potential drugs with anti-inflammatory activity, analgesic activity and lower ulcerogenic potential (Kumar *et al.*, 2008). A series of benzofuran/benzothiophene biphenyl oxo-acetic acids act as potent inhibitors of protein tyrosine phosphatase 1B with good oral antihyperglycemic activity (Malamas *et al.*, 2000). In this paper we report the crystal structure of the title compound, (I).

In the crystal of the title compound (Fig. 1), two carboxyl groups form a pair of hydrogen bonds in cyclic *R*_2_^2^(8) arrangement (Etter, 1990). The pairs of hydrogen bonds link the molecules into inversion dimers. The dimers are arranged in a herringbone pattern with an angle of 66.15 (1)°. The adjacent dimers are linked *via* C—H···π interactions with the H···π distance of 2.86 Å (Fig. 2). Some crystal structures containing biphenyl moiety have been reported. The two benzene rings are usually nearly coplanar for the biphenyl compounds without 2-substituents (Ali *et al.*, 2008; Cao, 2009; Margraf *et al.*, 2009; Li *et al.*, 2009; Charbonneau & Delugeard, 1977). But the title compound displays a twisted conformation with a dihedral angle of 47.51 (4)° between the phenyl and benzene planes. Planar conformations will be adopted by biphenyl compounds in the ground states. It is the crystal packing forces that produce the planar conformations for the biphenyl compounds (Brett *et al.*, 1999).

### Experimental

The crystals of (biphenyl-4-yloxy)acetic acid were unexpectedly obtained in the preparation of (biphenyl-4-yloxy)acetic acid-*β*-cyclodextrin inclusion complex. The experiment scheme is as follows: An ethanol solution of (biphenyl-4-yloxy)acetic acid (1 mmol, 5 ml) was added dropwise to an aqueous solution of *β*-cyclodextrin (1 mmol, 50 ml) and stirred at 50 °C for 6 h. The resulting solution was filtered and then stored at 40 °C. Colorless crystals suitable for the single X-ray diffraction were obtained after one week.

### Refinement

H atoms bonded to C were positioned geometrically with aromatic C—H = 0.93 Å and aliphatic C—H = 0.97 Å. Their displacement parameters were set at *U*_iso_(H) = 1.2*U*_eq_(C). The hydroxyl H atom was found in a Fourier map and refined with the constraint of O—H = 0.82 Å, *U*_iso_(H) = 1.2*U*_eq_(O).

### Crystal data

**Table:**

C_14_H_12_O_3_	*F*(000) = 480
*M**_r_* = 228.24	*D*_x_ = 1.380 Mg m^−^^3^
Monoclinic, *P*2_1_/*n*	Cu *K*α radiation, λ = 1.54178 Å
Hall symbol: -P 2yn	Cell parameters from 11208 reflections
*a* = 5.9118 (1) Å	θ = 4.6–76.4°
*b* = 28.5786 (3) Å	µ = 0.79 mm^−^^1^
*c* = 6.9017 (1) Å	*T* = 293 K
β = 109.631 (2)°	Plate, colourless
*V* = 1098.27 (3) Å^3^	0.43 × 0.42 × 0.40 mm
*Z* = 4	

### Data collection

**Table:**

Bruker SMART CCD area-detector diffractometer	2306 independent reflections
Radiation source: fine-focus sealed tube	2223 reflections with *I* > 2σ(*I*)
graphite	*R*_int_ = 0.028
φ and ω scans	θ_max_ = 77.0°, θ_min_ = 6.2°
Absorption correction: multi-scan (*SADABS*; Bruker, 2000)	*h* = −7→7
*T*_min_ = 0.727, *T*_max_ = 0.742	*k* = −36→35
11989 measured reflections	*l* = −7→8

### Refinement

**Table:**

Refinement on *F*^2^	Primary atom site location: structure-invariant direct methods
Least-squares matrix: full	Secondary atom site location: difference Fourier map
*R*[*F*^2^ > 2σ(*F*^2^)] = 0.047	Hydrogen site location: inferred from neighbouring sites
*wR*(*F*^2^) = 0.139	H-atom parameters constrained
*S* = 1.12	*w* = 1/[σ^2^(*F*_o_^2^) + (0.0818*P*)^2^ + 0.2839*P*] where *P* = (*F*_o_^2^ + 2*F*_c_^2^)/3
2306 reflections	(Δ/σ)_max_ < 0.001
155 parameters	Δρ_max_ = 0.21 e Å^−^^3^
0 restraints	Δρ_min_ = −0.23 e Å^−^^3^
0 constraints	

### Fractional atomic coordinates and isotropic or equivalent isotropic displacement parameters (Å^2^)

**Table:**

	*x*	*y*	*z*	*U*_iso_*/*U*_eq_	
O3	0.54080 (16)	0.06250 (3)	0.05489 (14)	0.0328 (3)	
O2	0.39398 (17)	0.02975 (3)	−0.32997 (15)	0.0364 (3)	
O1	0.76170 (18)	0.00213 (4)	−0.29161 (15)	0.0380 (3)	
H1	0.6955	−0.0065	−0.4105	0.057*	
C8	0.4590 (2)	0.11281 (5)	0.2933 (2)	0.0321 (3)	
H8	0.2999	0.1139	0.2069	0.039*	
C3	0.6254 (2)	0.08550 (4)	0.24154 (19)	0.0303 (3)	
C7	0.5320 (2)	0.13840 (4)	0.4743 (2)	0.0321 (3)	
H7	0.4203	0.1565	0.5085	0.039*	
C6	0.7706 (2)	0.13749 (4)	0.6066 (2)	0.0302 (3)	
C5	0.9320 (2)	0.10893 (5)	0.5536 (2)	0.0335 (3)	
H5	1.0905	0.1073	0.6411	0.040*	
C2	0.7191 (2)	0.04138 (5)	−0.0118 (2)	0.0328 (3)	
H2A	0.7931	0.0156	0.0790	0.039*	
H2B	0.8430	0.0641	−0.0063	0.039*	
C9	0.8549 (2)	0.16649 (4)	0.7961 (2)	0.0309 (3)	
C1	0.6092 (2)	0.02357 (4)	−0.2277 (2)	0.0314 (3)	
C4	0.8611 (2)	0.08296 (5)	0.3732 (2)	0.0342 (3)	
H4	0.9711	0.0640	0.3408	0.041*	
C11	1.1469 (3)	0.22017 (5)	1.0158 (2)	0.0403 (3)	
H11	1.2858	0.2379	1.0417	0.048*	
C10	1.0651 (2)	0.19293 (5)	0.8395 (2)	0.0351 (3)	
H10	1.1512	0.1923	0.7489	0.042*	
C13	0.8131 (3)	0.19494 (5)	1.1128 (2)	0.0379 (3)	
H13	0.7290	0.1954	1.2051	0.045*	
C14	0.7289 (2)	0.16808 (5)	0.9348 (2)	0.0335 (3)	
H14	0.5876	0.1510	0.9079	0.040*	
C12	1.0218 (3)	0.22096 (5)	1.1534 (2)	0.0399 (3)	
H12	1.0777	0.2389	1.2725	0.048*	

### Atomic displacement parameters (Å^2^)

**Table:**

	*U*^11^	*U*^22^	*U*^33^	*U*^12^	*U*^13^	*U*^23^
O3	0.0336 (5)	0.0349 (5)	0.0283 (5)	0.0004 (4)	0.0085 (4)	−0.0068 (4)
O2	0.0357 (5)	0.0383 (5)	0.0318 (5)	0.0054 (4)	0.0067 (4)	−0.0019 (4)
O1	0.0385 (5)	0.0448 (6)	0.0299 (5)	0.0059 (4)	0.0104 (4)	−0.0061 (4)
C8	0.0306 (6)	0.0336 (6)	0.0309 (7)	−0.0010 (5)	0.0084 (5)	−0.0019 (5)
C3	0.0363 (7)	0.0274 (6)	0.0267 (6)	−0.0019 (5)	0.0100 (5)	−0.0024 (4)
C7	0.0333 (6)	0.0314 (6)	0.0325 (7)	0.0011 (5)	0.0120 (5)	−0.0018 (5)
C6	0.0344 (6)	0.0268 (6)	0.0295 (6)	−0.0010 (5)	0.0106 (5)	−0.0005 (5)
C5	0.0325 (6)	0.0337 (6)	0.0317 (7)	0.0014 (5)	0.0074 (5)	−0.0034 (5)
C2	0.0341 (6)	0.0342 (6)	0.0294 (6)	0.0013 (5)	0.0097 (5)	−0.0043 (5)
C9	0.0351 (6)	0.0264 (6)	0.0296 (6)	0.0024 (5)	0.0089 (5)	−0.0004 (5)
C1	0.0367 (7)	0.0285 (6)	0.0287 (6)	0.0017 (5)	0.0106 (5)	0.0011 (5)
C4	0.0355 (7)	0.0324 (6)	0.0339 (7)	0.0041 (5)	0.0107 (5)	−0.0030 (5)
C11	0.0404 (7)	0.0360 (7)	0.0398 (8)	−0.0042 (6)	0.0072 (6)	−0.0056 (6)
C10	0.0358 (7)	0.0335 (6)	0.0350 (7)	−0.0006 (5)	0.0107 (6)	−0.0021 (5)
C13	0.0517 (8)	0.0333 (7)	0.0293 (7)	0.0033 (6)	0.0146 (6)	0.0000 (5)
C14	0.0398 (7)	0.0293 (6)	0.0318 (7)	−0.0004 (5)	0.0123 (5)	−0.0004 (5)
C12	0.0515 (8)	0.0326 (7)	0.0301 (7)	0.0004 (6)	0.0064 (6)	−0.0055 (5)

### Geometric parameters (Å, °)

**Table:**

O3—C3	1.3816 (15)	C2—C1	1.5002 (17)
O3—C2	1.4190 (16)	C2—H2A	0.9700
O2—C1	1.2427 (16)	C2—H2B	0.9700
O1—C1	1.2848 (16)	C9—C14	1.3971 (18)
O1—H1	0.8200	C9—C10	1.3982 (19)
C8—C7	1.3852 (18)	C4—H4	0.9300
C8—C3	1.3933 (18)	C11—C12	1.386 (2)
C8—H8	0.9300	C11—C10	1.3873 (19)
C3—C4	1.3862 (19)	C11—H11	0.9300
C7—C6	1.3996 (19)	C10—H10	0.9300
C7—H7	0.9300	C13—C12	1.386 (2)
C6—C5	1.3946 (18)	C13—C14	1.3914 (19)
C6—C9	1.4858 (17)	C13—H13	0.9300
C5—C4	1.3880 (18)	C14—H14	0.9300
C5—H5	0.9300	C12—H12	0.9300
			
C3—O3—C2	115.42 (10)	C14—C9—C6	121.53 (12)
C1—O1—H1	109.5	C10—C9—C6	120.07 (12)
C7—C8—C3	119.62 (12)	O2—C1—O1	125.12 (12)
C7—C8—H8	120.2	O2—C1—C2	122.36 (12)
C3—C8—H8	120.2	O1—C1—C2	112.52 (11)
O3—C3—C4	123.75 (12)	C3—C4—C5	119.61 (12)
O3—C3—C8	116.11 (11)	C3—C4—H4	120.2
C4—C3—C8	120.14 (12)	C5—C4—H4	120.2
C8—C7—C6	121.25 (12)	C12—C11—C10	120.08 (14)
C8—C7—H7	119.4	C12—C11—H11	120.0
C6—C7—H7	119.4	C10—C11—H11	120.0
C5—C6—C7	117.92 (12)	C11—C10—C9	120.93 (13)
C5—C6—C9	120.04 (12)	C11—C10—H10	119.5
C7—C6—C9	122.03 (11)	C9—C10—H10	119.5
C4—C5—C6	121.42 (12)	C12—C13—C14	120.31 (13)
C4—C5—H5	119.3	C12—C13—H13	119.8
C6—C5—H5	119.3	C14—C13—H13	119.8
O3—C2—C1	110.21 (11)	C13—C14—C9	120.52 (13)
O3—C2—H2A	109.6	C13—C14—H14	119.7
C1—C2—H2A	109.6	C9—C14—H14	119.7
O3—C2—H2B	109.6	C11—C12—C13	119.75 (13)
C1—C2—H2B	109.6	C11—C12—H12	120.1
H2A—C2—H2B	108.1	C13—C12—H12	120.1
C14—C9—C10	118.40 (12)		
			
C2—O3—C3—C4	−9.49 (18)	O3—C2—C1—O2	3.58 (18)
C2—O3—C3—C8	169.80 (11)	O3—C2—C1—O1	−176.89 (11)
C7—C8—C3—O3	−177.55 (11)	O3—C3—C4—C5	177.22 (11)
C7—C8—C3—C4	1.77 (19)	C8—C3—C4—C5	−2.0 (2)
C3—C8—C7—C6	0.2 (2)	C6—C5—C4—C3	0.4 (2)
C8—C7—C6—C5	−1.8 (2)	C12—C11—C10—C9	0.9 (2)
C8—C7—C6—C9	176.93 (12)	C14—C9—C10—C11	−0.1 (2)
C7—C6—C5—C4	1.5 (2)	C6—C9—C10—C11	−179.91 (12)
C9—C6—C5—C4	−177.23 (12)	C12—C13—C14—C9	0.8 (2)
C3—O3—C2—C1	−172.07 (10)	C10—C9—C14—C13	−0.80 (19)
C5—C6—C9—C14	−133.31 (14)	C6—C9—C14—C13	179.04 (12)
C7—C6—C9—C14	48.02 (18)	C10—C11—C12—C13	−0.8 (2)
C5—C6—C9—C10	46.52 (18)	C14—C13—C12—C11	0.0 (2)
C7—C6—C9—C10	−132.15 (14)		

### Hydrogen-bond geometry (Å, °)

**Table:**

Cg is the centroid of the C9–C14 ring.

**Table:**

*D*—H···*A*	*D*—H	H···*A*	*D*···*A*	*D*—H···*A*
O1—H1···O2^i^	0.82	1.81	2.6235 (13)	169
C12—H12···Cg^ii^	0.93	2.86	3.6392 (16)	142

Symmetry codes: (i) −*x*+1, −*y*, −*z*−1; (ii) *x*+1/2, −*y*+1/2, *z*+1/2.
